# Supplementary material for: Hierarchical true prevalence, risk factors and clinical symptoms of tuberculosis among suspects in Bangladesh
Source: PLoS One. 2022 Jul 12;17(7):e0262978. doi: 10.1371/journal.pone.0262978 (PMC9275716; doi:10.1371/journal.pone.0262978)
Supplement: S5 File — (DOCX) [file pone.0262978.s005.docx]

**Univariable association of demographic and other risk factors with human tuberculosis (hTB) in Mymensingh, Sirajganj and Dhaka districts, Bangladesh (n=684)**

| **Variables** | **Category** | **hTB Negative** | **hTB Positive (%)** | **Estimate (SD)** | **Odds ratio**  **(95% PI)** | **Bayesian P-value** | **n_eff** | **RHat** |
| --- | --- | --- | --- | --- | --- | --- | --- | --- |
| Age groups (years) | ≤25 | 126 | 15 (10.6) | 0.65 (0.48) | 1.9 (0.76;5.16) | 0.08 | 27,349 | 1 |
|  | >25-45 | 232 | 32 (12.1) | 0.81 (0.44) | 2.24(0.98;5.59) | 0.02 | 26,518 | 1 |
|  | >45-60 | 145 | 27 (15.7) | 1.11 (0.45) | 3.02(1.29;7.67) | 0.004 | 27,235 | 1 |
|  | >60 | 101 | 6 (5.6) | Reference | 1 |  | - | - |
| Sex | Male | 403 | 49 (10.8) | Reference | 1 |  | - | - |
|  | Female | 201 | 31 (13.4) | 0.24 (0.24) | 1.3 (0.77,2.01) | 0.17 | 63,498 | 1 |
| Education | Below SSC | 442 | 59 (11.8) | -0.003 (0.49) | 0.99 (0.07;2.58) | 0.44 | 29,553 | 1 |
|  | SSC and above | 162 | 21 (11.5) | Reference | 1 |  | 28,978 | 1 |
| Occupation | Business | 69 | 12 (14.8) | 0.64 (0.63) | 1.90 (0.57, 7.29) | 0.15 | 18,375 | 1 |
|  | Day labor | 30 | 10 (25.0) | 1.30 (0.68) | 3.66 (1.01, 14.59) | 0.02 | 17,980 | 1 |
|  | Farmer | 133 | 14 (9.5) | 0.17 (0.64) | 1.18 (0.36, 4.37) | 0.40 | 17,297 | 1 |
|  | Housewife | 174 | 23 (11.7) | 0.41 (0.60) | 1.50 (0.48, 5.50) | 0.25 | 15,972 | 1 |
|  | Service | 111 | 11 (9.0) | 0.09 (0.65) | 1.09 (0.32, 4.23) | 0.44 | 18,798 | 1 |
|  | Students | 61 | 8 (11.6) | 0.35 (0.69) | 1.41 (0.37, 5.68) | 0.29 | 21,190 | 1 |
|  | Minor and old people (>70 yrs) | 26 | 2 (7.1) | Reference | 1 |  |  | 1 |
| Residence | Rural | 394 | 48 (10.9) | Reference | 1 |  | - | - |
|  | Urban | 210 | 32 (13.2) | 0.24 (0.25) | 1.27 (0.77; 2.08) | 0.17 | 52,020 | 1 |
| Smoking | Yes | 322 | 48 (13.0) | 0.28 (0.24) | 1.32 (0.82;2.13) | 0.12 | 26,645 | 1 |
|  | No | 282 | 32 (10.2) | 0.28 (0.25) | 1 |  | - | - |
| Presence of TB patient in the family or neighborhood | Yes | 35 | 32 (47.8) | 2.37 (0.29) | 10.77(6.07;19.16) | <0.001 | - | - |
|  | No | 569 | 48 (7.8) | Reference | 1 |  | - | - |
| Taking care of cattle | No | 479 | 71 (12.9) | 0.79 (0.39) | 2.19 (1.09; 4.81) | 0.012 | 53,097 | 1 |
|  | Yes | 125 | 9 (6.7) | Reference | 1 |  | - | - |
| Presence of cattle in the family | No | 451 | 63 (12.3) | 0.27 (0.29) | 1.31 (0.75;2.36) | 0.18 | 60,230 | 1 |
|  | Yes | 153 | 17 (10.0) | Reference | 1 |  | - | - |
| Handling raw milk or meat | Yes | 576 | 77 (11.8) | 0.28 (0.65) | 1.32 (0.42, 5.38) | 0.65 | 50,592 | 1 |
|  | No | 28 | 3 (9.7) | Reference | 1 |  | - | - |
| Drinking raw milk | Yes | 20 | 1(4.8) | Reference | 1 |  |  |  |
|  | No | 584 | 79 (11.9) | 1.09 (1.02) | 2.99 (0.54;30.66) | 0.13 | 39,633 | 1 |
