# Supplementary material for: Hierarchical true prevalence, risk factors and clinical symptoms of tuberculosis among suspects in Bangladesh
Source: PLoS One. 2022 Jul 12;17(7):e0262978. doi: 10.1371/journal.pone.0262978 (PMC9275716; doi:10.1371/journal.pone.0262978)
Supplement: S6 File — (DOCX) [file pone.0262978.s006.docx]

**Clinical symptoms and comorbidities associated with human tuberculosis (hTB) based on univariable logistic regression analyses (n=684)**

| **Variable** | **Category** | **hTB Negative** | **hTB Positive (%)** | **Estimate (SD)** | **Odds ratio (95% CrI)** | **Bayesian P-value** | **N_eff** |
| --- | --- | --- | --- | --- | --- | --- | --- |
| Fever | Yes | 479 | 78 (14.0) | 2.3 (0.7) | 9.9 (3.3; 44.7) | <0.001 | 23,861 |
|  | No | 125 | 2 (1.6) | Reference | 1 |  | - |
| Cough | Yes | 530 | 80 (13.1) | 3.6 (1.3) | 37.4 (4.4; 790.7) | <0.001 | 16,653 |
|  | No | 74 | 0 (0) | Reference | 1 |  | - |
| Fatigue | Yes | 44 | 29 (39.7) | 1.9 (0.3) | 7.3 (4.2; 12.9) | <0.001 | 31,096 |
|  | No | 560 | 51 (8.3) |  | 1 |  | - |
| Weight loss | Yes | 34 | 26 (43.3) | 2.1 (0.3) | 8.1 (4.4; 14.7) | <0.001 | 41,145 |
|  | No | 570 | 54 (8.7) | Reference | 1 |  | - |
| Chest pain | Yes | 22 | 6 (21.4) | 0.7 (0.5) | 2.0 (0.73; 5.0) | 0.08 | 33,688 |
|  | No | 582 | 74 (11.3) | Reference | 1 |  | - |
| Hemoptysis | Yes | 12 | 13 (52.0) | 2.3 (0.4) | 9.5 (4.1; 22.5) | <0.001 | 34,489 |
|  | No | 592 | 67 (10.2) | Reference | 1 |  | - |
| Night sweats | Yes | 5 | 9 (64.3) | 2.7 (0.6) | 14.6 (4.9; 47.0) | <0.001 | 42,035 |
|  | No | 599 | 71 (10.6) | Reference | 1 |  | - |
| Comorbidities (overall) | Yes | 62 | 5 (7.5) | Reference | 1 |  | - |
|  | No | 542 | 75 (12.2) | 0.6 (0.5) | 1.8 (0.8, 5.1) | 0.09 | 34,382 |

CrI: Credible Interval, N_eff=Effective sample size, SD=Standard deviation, RHat=1 for all parameters
