# Supplementary material for: Hierarchical true prevalence, risk factors and clinical symptoms of tuberculosis among suspects in Bangladesh
Source: PLoS One. 2022 Jul 12;17(7):e0262978. doi: 10.1371/journal.pone.0262978 (PMC9275716; doi:10.1371/journal.pone.0262978)
Supplement: S7 File — (DOCX) [file pone.0262978.s007.docx]

| Risk factors | Category | Odds ratio | | Changes (%) |
| --- | --- | --- | --- | --- |
|  |  | With smoking | Without smoking |  |
| Age (years) |  |  |  |  |
|  | ≤ 25 | 1.79 | 1.47 | 21.7% |
|  | >25to 45 | 2.67 | 2.44 | 9.4% |
|  | > 45 to 60 | 3.42 | 3.15 | 8.6% |
|  | > 60 | Reference | Reference |  |
| Tuberculosis patient in the family or neighborhood |  |  |  |  |
|  | Yes | 12.30 | 11.79 | 4.3% |
|  | No | Reference | Reference |  |

Supplementary file 7: Changes in the odds ratio after adding smoking in the final multivariable model
