# Supplementary material for: Hierarchical true prevalence, risk factors and clinical symptoms of tuberculosis among suspects in Bangladesh
Source: PLoS One. 2022 Jul 12;17(7):e0262978. doi: 10.1371/journal.pone.0262978 (PMC9275716; doi:10.1371/journal.pone.0262978)
Supplement: S8 File — (PDF) [file pone.0262978.s008.pdf]

Status report:

The report will become available below

tPRiors-dynamic-report

20 May, 2022

General information

This report has automatically been generated by the shiny web-application tPRiors as an R Markdown document based on your data input and prior selection. The web-application can be found at https://publicandonehealth.shinyapps.io/tPRiors (https://publicandonehealth.shinyapps.io/tPRiors). We advice users that after observing the results of this report to avoid re-updating their prior beliefs to avoid hampering the credibility of these results.

The following section describes your input. During set-up the user assumed that:

1. Multiple modelled,
2. No, zero prevalence was modeled and .
3. between the Apparent and True prevalence the True prevalence was modelled and
4. (the) Mean was used to elicitate prior knowledge.

If the true prevalence (inline equation test: (π_t) is modelled the following relation is utilized to acquire its posterior distribution, inline equation test: $\pi_a = \pi_t \cdot S_e(1-\pi_t) \cdot (1-S_p)$, where inline equation test: S_p, S_e denotes the specificity and sensitivity of the diagnostic test and inline equation test: π_a the apparent prevalence.

The elicited prevalence prior

The selected prior distribution of the True prevalence has the following descriptive characteristics and density plot.

```
## [1] "Summary of True prevalence Beta(0.1671,0.6687) prior"
```

```
##      Min.   1st Qu.    Median      Mean   3rd Qu.      Max.
## 0.0000000 0.0005439 0.0311828 0.2033831 0.3180634 0.9999750
```

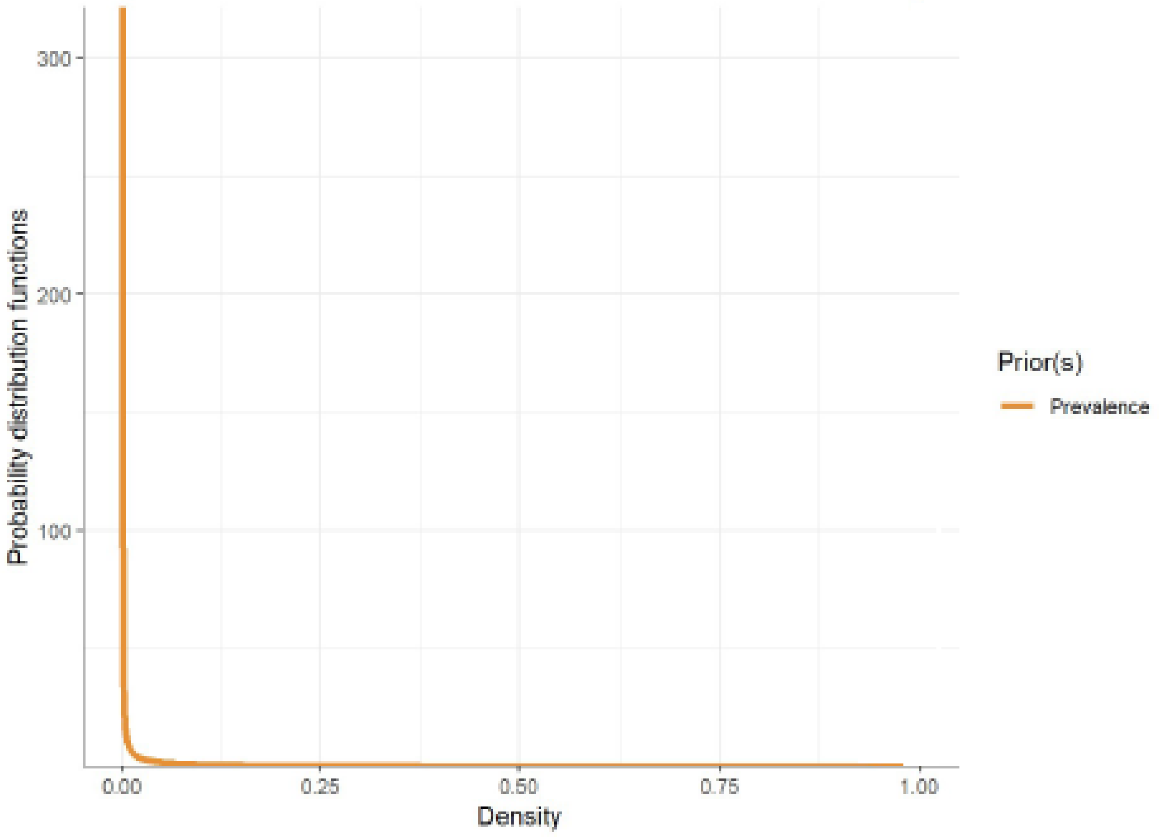

The elicited Specificity/Sensitivity priors

The selected prior distribution of the Sensitivity and Specificity have the following descriptive characteristics and density plot.

```
## [1] "Summary of sensitivity Beta(16.4,8.83) prior"
```

```
##    Min. 1st Qu.  Median    Mean 3rd Qu.    Max. 
##  0.2758  0.5852  0.6530  0.6483  0.7148  0.9398
```

```
## [1] "Summary of specificity Beta(34.11,1.42) prior"
```

```
##    Min. 1st Qu.  Median    Mean 3rd Qu.    Max. 
##  0.7257  0.9451  0.9684  0.9601  0.9842  0.9999
```

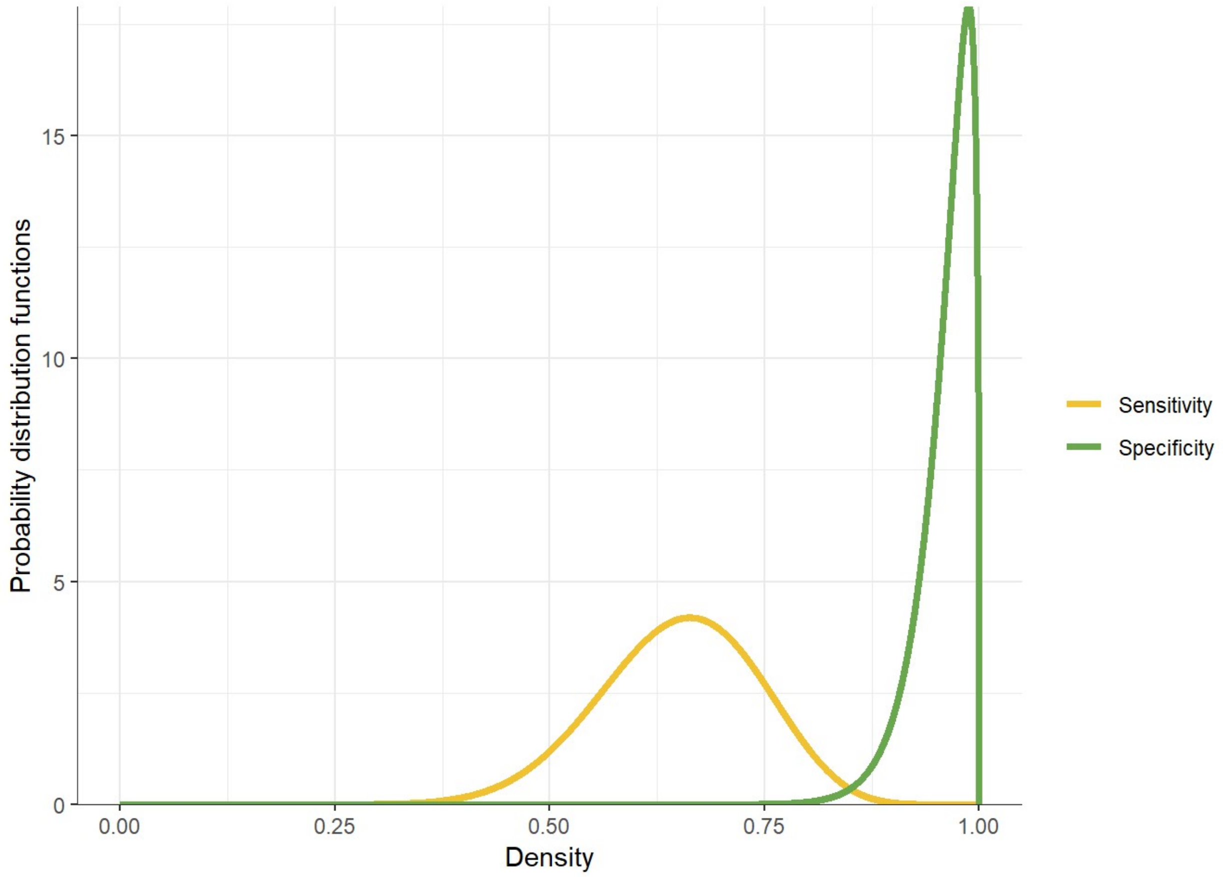

The data

A summary of the input data is provided below

```
## [1] "If modelled, the assumed mean probability for non-zero prevalence was set equal to NA"
```

The model

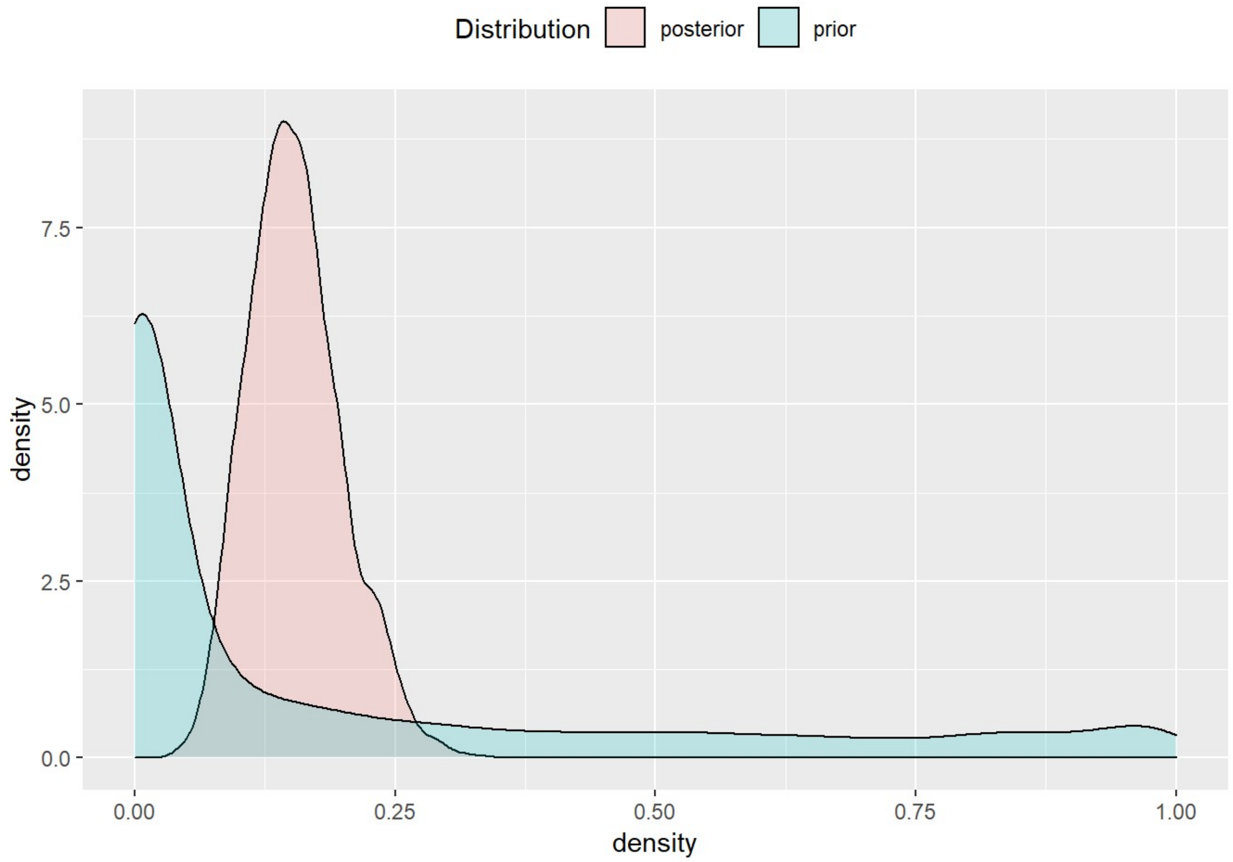


```
## [1] "Summary of posterior prevalence"
```

```
##    Min. 1st Qu.  Median    Mean 3rd Qu.    Max.
##  0.0372  0.1222  0.1499  0.1541  0.1812  0.3355
```

```
## [1] "Quantiles of posterior prevalence"
```

```
##      0%      1%    2.5%     10%     20%     50%     80%     90%   97.5%     99%    100%
## 0.03720 0.06778 0.07590 0.09834 0.11535 0.14985 0.19053 0.21630 0.25135 0.27042 0.33547
```

```
## [1] "The probability that the posterior prevalence is higher than 0.05 equals to"
```

```
## [1] 0.997
```

Study-level boxplot and numerical summaries of posterior prevalence samples

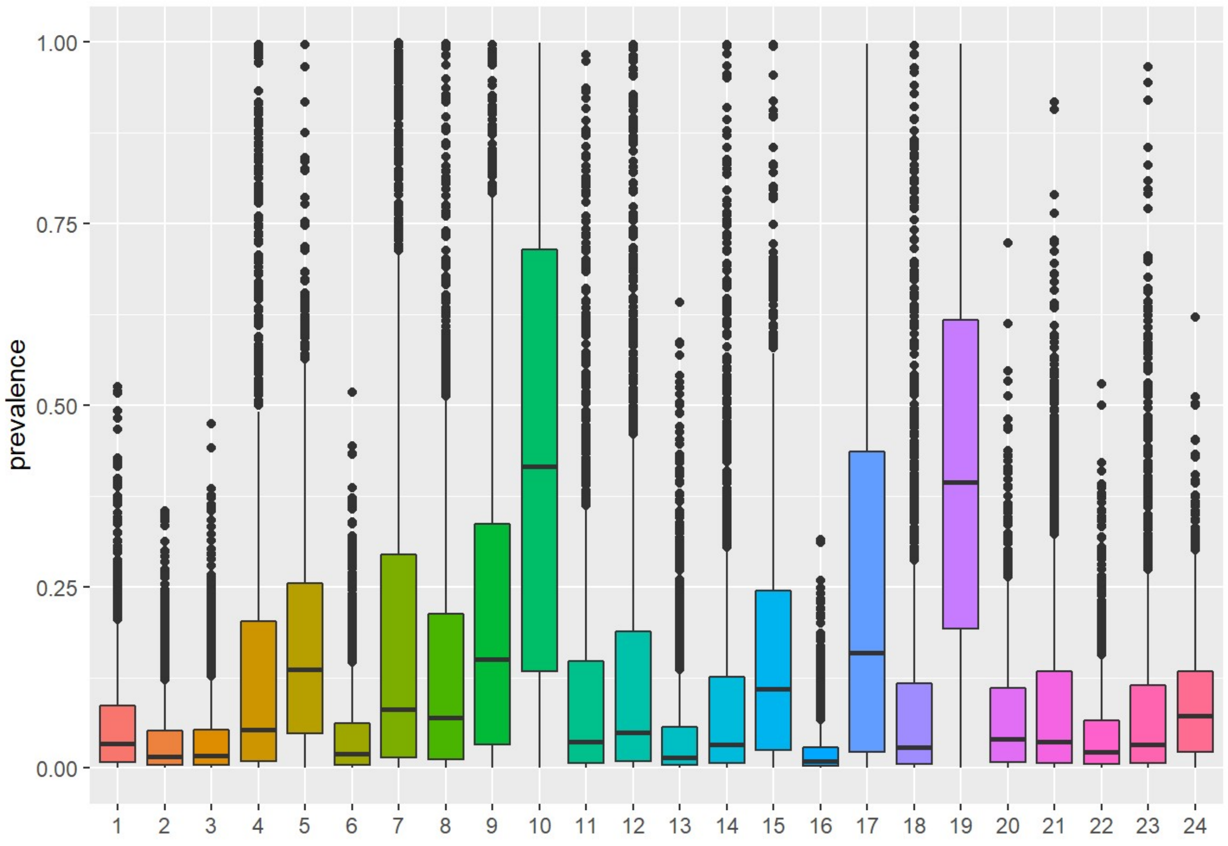


```
##    T_prev_mean   LL95  UL95 T_prev_median  Q025  Q975
## 1        0.063  0.002 0.123         0.034 0.001 0.262
## 2        0.040 -0.030 0.110         0.016 0.001 0.205
## 3        0.042 -0.016 0.099         0.017 0.001 0.215
## 4        0.144 -0.099 0.388         0.053 0.001 0.789
## 5        0.172  0.055 0.289         0.136 0.002 0.557
## 6        0.048 -0.023 0.119         0.020 0.001 0.239
## 7        0.196 -0.122 0.514         0.081 0.001 0.878
## 8        0.146 -0.046 0.339         0.070 0.001 0.666
## 9        0.218  0.009 0.426         0.150 0.002 0.802
## 10       0.438  0.003 0.873         0.416 0.002 0.984
## 11       0.113 -0.094 0.320         0.037 0.001 0.659
## 12       0.141 -0.100 0.382         0.049 0.001 0.798
## 13       0.051 -0.079 0.181         0.015 0.001 0.317
## 14       0.106 -0.164 0.376         0.032 0.001 0.629
## 15       0.161  0.027 0.295         0.109 0.002 0.590
## 16       0.024 -0.012 0.059         0.009 0.001 0.132
## 17       0.266 -0.167 0.700         0.158 0.001 0.947
## 18       0.100 -0.140 0.340         0.029 0.001 0.629
## 19       0.418  0.150 0.687         0.394 0.004 0.969
## 20       0.073  0.007 0.139         0.040 0.001 0.300
## 21       0.098 -0.043 0.240         0.037 0.001 0.483
## 22       0.049 -0.016 0.114         0.022 0.001 0.238
## 23       0.092 -0.035 0.218         0.033 0.001 0.519
## 24       0.090  0.041 0.139         0.072 0.002 0.295
```

Predictions

Predictive distribution of y for a single new study with $n = 100$. (Unstable for less than 10 clusters)

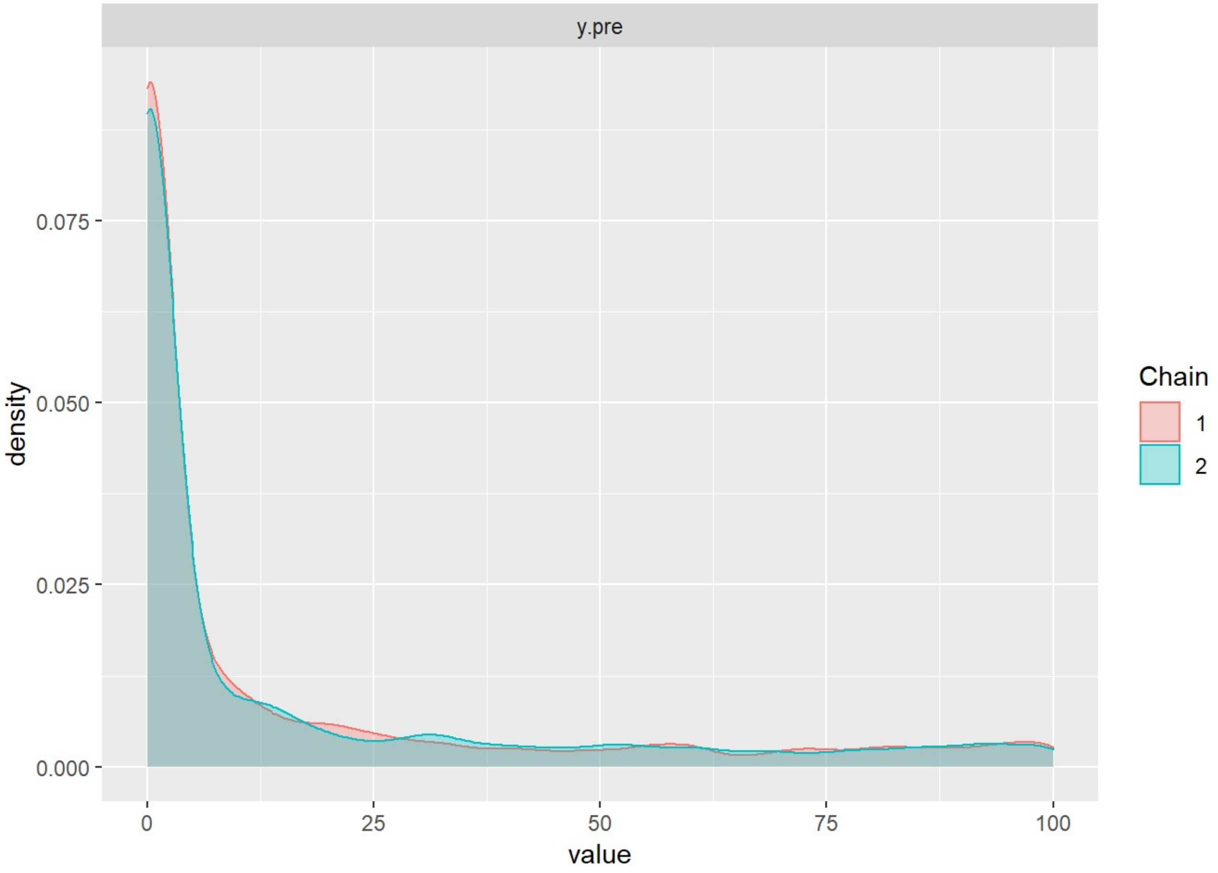

The predictive prevalence mean of a future study equals to (Unstable for less than 10 clusters)

```
## [1] 0.155
```

The probability that the prevalence equals to 0 (zero) equals to

```
## [1] 0
```

Diagnostics

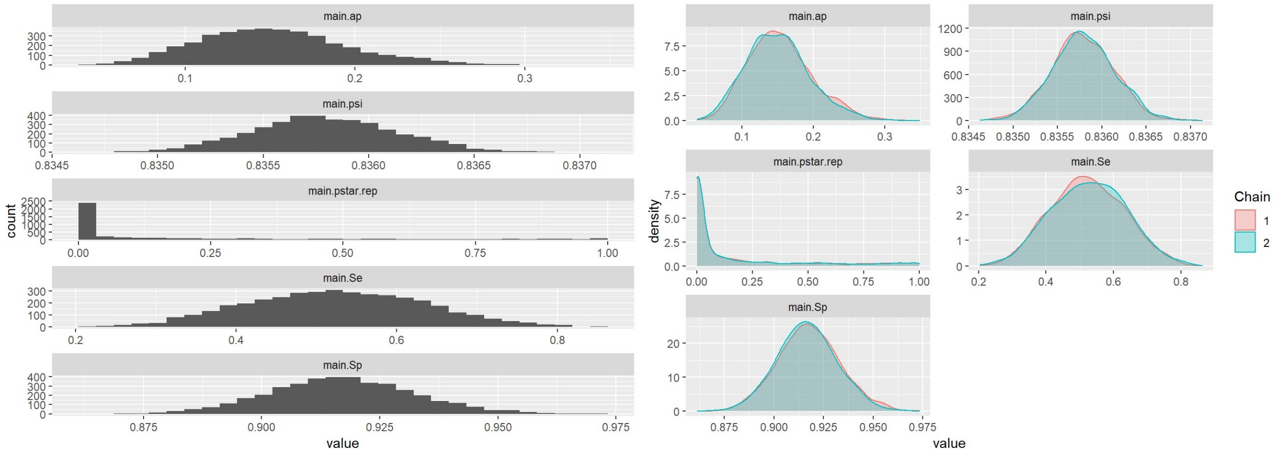

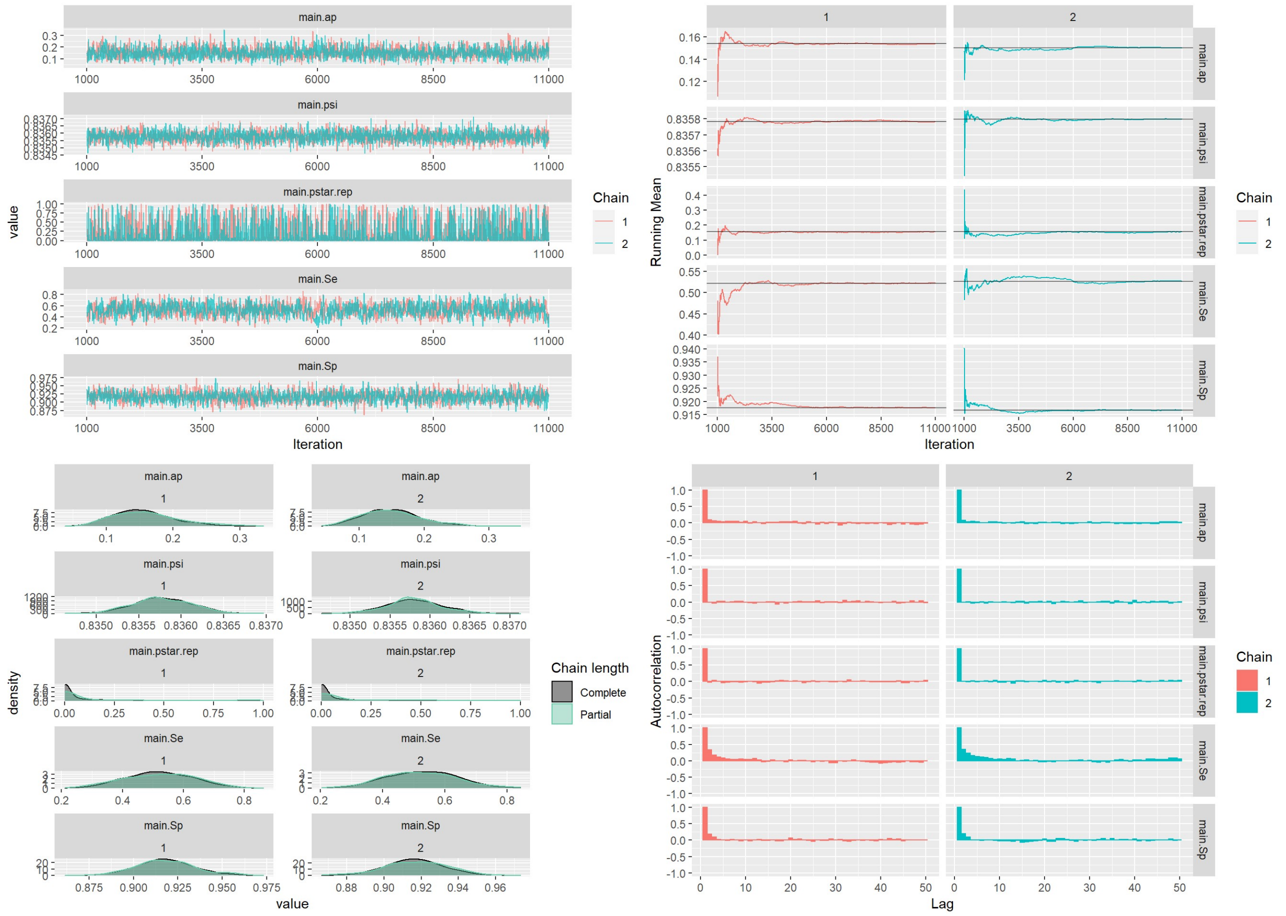

End of tPRiors report.

Instructions for using the resulted .rData files

Users can further access the analysis with the following .rData files. We provide below three files under the tabs; 1) Input, 2) Model and 3) Output. The user should first open R or Rstudio and then search and open the .rData file of interest through the tab or through the load function. The input data in R form can be loaded by "load("~/…/InputData.RData")". The final JAGS model can be loaded in R via "load("~/…/JagsModel.rData")", while the MCMC samples can be directly loaded in R via "load(~/…/Model1.mcmc.RData), where"…" stands for the local directory path where the file of interest has been placed.

Working with the <Model.mcmc> object

The Model.mcmc object contains MCMC samples produced by the current Bayesian analysis set-up. When loaded in R the user can perform further descriptive analysis and check more diagnostics, which is especially important in multiple populations where the number of parameters becomes very large and inference may become unstable. We provide a number of potentially useful descriptive and diagnostic plots from the CODA package alone or in combination with the ggmcmc R package.

traceplot(Model1.mcmc)

autocorr.plot(Model1.mcmc)

geweke.plot(Model1.mcmc)

When working with multiple population models that contain many parameters it can be convinient to use the family option of the ggs function group. First load the data files, then install and load the ggmcmc library. In the

case of multiple populations if family="main" then, only main parameters are being plotted, if family="sub" then only study parameters are selected, while if family="pre" then predictive parameters are selected and returned. Some examples are provided below.

S2<-ggs(Model1.mcmc)

ggs_histogram(S2,family = "main") # Histograms (Main)

ggs_density(S2,family = "main") # Density plots for main parameters

ggs_density(S2,family = "sub") # Density plots for study prevalences

ggs_density(S2,family = "pre") # Density plots for predictive parameters

ggs_traceplot(S2,family = "main") # Trace plots (Main)

ggs_running(S2,family = "main") # Running means plots (Main)

ggs_compare_partial(S2,family = "main") # Partial chain comparison plots (Main)

ggs_autocorrelation(S2,family = "main") # Autocorrelation plots (Main)

Input Model Output

Download data (session/61ef3c25613123b7c67c28d2853a88db/download/downloadData?w=)
